# What Art Has Done for Dentistry—and What Dentistry Has Done for Art

**Published:** 1902-11-15

**Authors:** B. J. Cigrand

**Affiliations:** Chicago


### WHAT ART HAS DONE FOR DENTISTRY—AND WHAT DENTISTRY HAS DONE FOR ART.

Abstract of a paper read by Dr. B. J. Cigrand, of Chicago, before the Southwestern Michigan Dental Society, and which will be published in full in the *Items of Interest* at an early date.

The essayist referred to a paper read before the Chicago Odontographic Society—on "Dental Deformities in Fine Art and Sculpture"—in which he showed from engravings and photographs, that many of the most highly credited drawings were actually deformed as far as the dental organs and tissues were concerned.

This paper is a continuation of a study, and attempts to disclose the recognition of our profession by artists; and also to call attention to contributions our profession has made to art. Artists have found little to inspire them in our profession or even in dental features. Not more than six well known pictures possess any truly dental characteristics. The patient and skilful ministrations of the dentist, remain to be portrayed by a great artist.

In the Dresden Art Gallery is the well known painting by Gerard Don, entitled "*The Dentist.*" The dentist with pompous air and satisfied expression, holds up as a trophy the tooth just extracted, while a distressed looking boy, well displays the feelings of one required to endure such an operation. It is a good illustration of a dental operation in later mediaeval times.

Another picture in the same gallery is entitled "*A Successful Dental Operation.*" It illustrates the commotion usually attending such an operation. The peculiarity of this picture is that its illumination is from a single candle light.

In the gallery at Munich is the well known picture entitled "*A Bad Tooth.*" This is the picture showing an extraction in a royal household, and its attendant commotion. It is a perfect picture of an old time court dentist and his attendant, and clearly depicts the dread attending tooth extracting in those days. The artist is unknown.

The paper refers to the portrait of General Washington, known as the Peal portrait, which was painted by a dentist, Dr. Peal. This is the only portrait of Washington which does not picture him with a distorted mouth. Dr. Peal suggested the use of platinum in connection with porcelain teeth, and was one of the first to call attention to the variations in shades of the teeth, and made valuable suggestions to tooth manufacturers.

To restore the features of the face destroyed by loss of the teeth is indeed the crowning glory of prosthesis. To recognize the form and character of teeth required and to produce shades in harmony with the age and temperament of the patient is both an art and a science. It is always safe in selecting artificial teeth for crowns or partial sets to select a shade slightly deeper than the natural teeth with which it is to harmonize. Because the natural teeth darken with age, and the teeth are neverso clean as when matched by the dentist. It is also essential that accidental defects in the teeth should be harmonized in the artificial substitute, pits and irregularity of form should be reproduced in the porcelain.

A cultivation of the art side of prosthesis will result in a high order of skill in mechanical invention, resulting in better appliances.

Dr. Norman W. Kingsley, of New York City, and Dr. T. S. Hitchcock, of Oswego, N. Y., are brilliant illustrations of what dentistry has done to develope art in men who have given themselves up to art impulses, their art works are not only unique in character but judged by artistic standard only, they rank among the higher grades of portraiture.

A skillful mechanical dentist is not always—in fact rarely is—an artist. In manual training schools, boys who give great promise in the mechanical department very frequently fail utterly in the artistic or drawing departments.

It is one thing to produce an article from a design made by some one else; and quite another thing to make the design and reproduce it, or produce the design in the concrete form without the drawing on paper, from a conception indelibly fixed in the mind of the worker, as is illustrated in sculpture or carving.

In prosthesis of every branch the successful dentist of the future will be the one who cultivates art as well as mechanics not only in a broad sense, but in its application to the specialty which he adopts. The art features of the human face, characteristic temperaments as well as mechanical skill are to be mastered to reach the highest possibilities in practice.

